# Binary Communication with Gazeau–Klauder Coherent States

**DOI:** 10.3390/e22020201

**Published:** 2020-02-10

**Authors:** Jerzy Dajka, Jerzy Łuczka

**Affiliations:** 1 Institute of Physics, University of Silesia in Katowice, 40-007 Katowice, Poland; jerzy.luczka@us.edu.pl; 2 Institute of Computer Science, University of Silesia in Katowice, 40-007 Katowice, Poland; 3 Silesian Center for Education and Interdisciplinary Research, University of Silesia in Katowice, 40-007 Chorzów, Poland; 4 Institute of Mathematics, University of Silesia in Katowice, 40-007 Katowice, Poland

**Keywords:** Gazeau–Klauder coherent states, Helstrom bound

## Abstract

We investigate advantages and disadvantages of using Gazeau–Klauder coherent states for optical communication. In this short paper we show that using an alphabet consisting of coherent Gazeau–Klauder states related to a Kerr-type nonlinear oscillator instead of standard Perelomov coherent states results in lowering of the Helstrom bound for error probability in binary communication. We also discuss trace distance between Gazeau–Klauder coherent states and a standard coherent state as a quantifier of distinguishability of alphabets.

## 1. Introduction

Quantum optical implementations of quantum information processing, including communication and computation, seems to be one of the most promising kinds today [1]. It is related to the maturity of both theoretical and experimental techniques developed in the last hundred years. It was quite early when the Quantum Community recognized the usefulness of the ‘most classical’ among quantum states—the coherent states—in quantum information processing [1,2,3,4]. Even recently coherent states with a non-random phase, despite certain limitations [2], have found their application in the very hot branch of quantum communication related to quantum key distribution [5]. The idea is simply to utilize as an alphabet a pair of coherent states [1]

(1) $$\begin{array}{ccc} \rho_{0} & = & |0\rangle \langle 0|,\;\;\;\rho_{1}=|z\rangle \langle z|, \end{array}$$

where |z〉=D(z)|0〉 is a coherent state related to the vacuum state |0〉 via the displacement operator $D\left(z\right)=\exp\left(-za^{†}-\bar{z}a\right)$ representing the Heisenberg–Weyl algebra $[a,a^{†}]=1$ [6]. Let us notice that the apparent simplicity of that proposal is at a price of non-orthogonality of the ‘letters’, i.e., $\mathrm{tr}(\rho_{0}\rho_{1})\neq 0$, resulting in their limiting distinguishability. Since coherent states do not require nonlinear media for their generation it seems advantageous [3] to use them in comparison to, e.g., earlier proposals utilizing squeezed states [7] demanding ‘hard’ nonlinearity. However, recent progress in experimental techniques may reverse this trend at least in the cases when going beyond standard coherent states becomes advantageous. Using the Schrödinger cat states as candidates for orthogonal letters of alphabet states serves as an example [1].

The aim of this work is to present an example of a candidate for an alphabet consisting of Gazeau–Klauder coherent states [8]. We analyze binary communication with Gazeau–Klauder states related to an oscillator equipped with a polynomial nonlinearity typical for the Kerr media. The Gazeau–Klauder coherent states have been studied for a variety of quantum systems: A one-mode system with sinusoidal potential [9], systems characterized by the Pöschl-Teller [10] and Morse potentials [11], for nonlinear Kerr-type oscillators [12], quantum particles confined by a double–well potential [13] and pseudoharmonic oscillators [14]. They can also serve as a basis of a very natural generalization of the cat states [15,16]. Our aim is to expand a list of potential applications of Gazeau–Klauder construction to a class of communication problems utilizing the non-orthogonal binary alphabet formed by Gazeau–Klauder coherent states. We show that such a choice can result in lowering of the Helstrom bound for a receiver error that may balance an obvious disadvantage of using nonlinear systems leading to non-trivial Gazeau–Klauder coherent states. The paper is organized as follows: After providing a short review of the Gazeau–Klauder construction of coherent states for a quantum bosonic system with a Kerr-type polynomial nonlinearity we calculate, as a quantifier of distinguishability of states, trace distance between Gazeau–Klauder states for a nonlinear system and corresponding standard states in a linear limit. Further, we propose a binary communication scheme utilizing an alphabet consisting of two Gazeau–Klauder coherent states as an alternative for well established schemes utilizing standard (Perelomov) coherent states. For such a scheme we calculate the Helstrom bound minimizing (over all possible positive-operator-valued measurements (POVM)) the error in the receiver. In the last two sections we discuss and conclude our work.

## 2. Results

Standard coherent states [6] are most natural for harmonic potential systems exhibiting the Heisenberg–Weyl symmetry which is a first step toward generalized coherent states in the Gilmore-Perelomov sense [6] exhibiting different symmetries. However, in an absence of almost any symmetry it is still possible to construct a class of states equipped with most of the desired properties of coherent states: The Gazeau–Klauder coherent states [8] solely associated with Hamiltonians of systems under consideration.

For the sake of completeness, let us recall the construction proposed in reference [8]. Let *H* be a Hamiltonian of the system with purely discrete non-degenerate (either finite or infinite) spectrum. The first step in constructing the Gazeau–Klauder states is to solve the eigenvalue problem:

(2) $$\begin{array}{c} H|n\rangle =E_{n}|n\rangle \equiv \hbar \omega \varepsilon_{n}|n\rangle ,\;\;n=0,1,2,\ldots \end{array}$$

The Gazeau–Klauder coherent states |J,γ〉 are two-parameter states with real-valued J≥0 and γ∈(−∞,∞) defined by the relation [8]

(3) $$\begin{array}{c} |J,\gamma \rangle =\frac{1}{C(J)}\sum_{n=0}^{\infty }\frac{J^{n/2}\exp\left(-i\gamma e_{n}\right)}{\sqrt{\rho_{n}}}|n\rangle , \end{array}$$

where

(4) $$\begin{array}{c} e_{n}=\varepsilon_{n}-\varepsilon_{0}=\frac{E_{n}-E_{0}}{\hbar \omega },\;\rho_{n}=\Pi_{j=1}^{n}e_{j},\;C^{2}\left(J\right)=\sum_{n=0}^{\infty }\frac{J^{n}}{\rho_{n}} \end{array}$$

and $\rho_{0}=1$. The other parameters in Equation (3) can be equipped with a clear physical meaning [8]: (i) 〈J,γ|H|J,γ〉∼J corresponds to a mean energy of the system, and (ii) its phase γ is related to a temporal stability via exp(−iHt)|J,γ〉=|J,γ+ωt〉. As the maximal value of *J* is bounded from above by the radius of convergence of the series C(J), a choice of *J* leading to a well defined quantum state remains limited.

The most elementary generalization of the standard coherent states [6] leading to the Gazeau–Klauder coherent states of non-trivial properties is for a bosonic oscillator with a polynomial nonlinearity. Let us consider a nonlinear oscillator of the Kerr type studied in the context of Gazeau–Klauder states in reference [12]. It is described by the bosonic Hamiltonian

(5) $$\begin{array}{c} H=\hbar \omega a^{†}a+\hbar \chi a^{†2}a^{2}\equiv \hbar \omega \hat{N}+\hbar \chi \left({\hat{N}}^{2}-\hat{N}\right), \end{array}$$

where $a^{†}$ and *a* are the creation and annihilation boson operators, $\hat{N}=a^{†}a$ is a number operator and χ is related to the nonlinear susceptibility of the Kerr medium [12], i.e., a medium with a refraction index depending on the field intensity [17,18].

From Equation (5) it follows that the energy eigenvalues are given by

(6) $$\begin{array}{c} e_{n}=\varepsilon_{n}=n-\mu n+\mu n^{2},\;\;\;\;n=0,1,2\ldots \end{array}$$

where μ=χ/ω (typically not exceeding a range of unity μ∼1) is the susceptibility rescaled with respect to the bare oscillator energy and hence one can explicitly construct Gazeau–Klauder states with

(7) $$\begin{array}{ccc} \rho_{n} & = & \Gamma \left(n+1\right)\mu^{n}\Gamma \left(\frac{\mu \;n+1}{\mu }\right)/\Gamma \left(\mu^{-1}\right). \end{array}$$

Let us notice that $\rho_{n}=\Gamma \left(n+1\right)=n!$ for the harmonic oscillator, i.e., for μ=0. In this case the Gazeau–Klauder coherent states reduce to the standard coherent states for $z=\sqrt{J}$. Moreover, since eigenstates of the Kerr Hamiltonian (5) coincide with the eigenstates of the standard harmonic oscillator one can consider the Gazeau–Klauder states studied in this paper as a very first ’extension’ of the standard coherent states construction of Perelomov [6]. In particular, for γ=0, one gets

(8) $$\begin{array}{c} |J,0\rangle =\frac{1}{C(J)}\sum_{n=0}^{\infty }\frac{J^{n/2}}{\sqrt{\rho_{n}}}|n\rangle , \end{array}$$

with

(9) $$\begin{array}{ccc} C^{2}\left(J\right) & = & I_{\frac{1-\mu }{\mu }}\left(2\;\sqrt{\frac{J}{\mu }}\right)\Gamma \left(\mu^{-1}\right){\left(\frac{J}{\mu }\right)}^{-\frac{1-\mu }{2\mu }} \end{array}$$

expressed in terms of the modified Bessel function $I_{\alpha }\left(z\right)$. Further, as a potential alternative for the traditional choice given by Equation (1), here we consider

(10) $$\begin{array}{ccc} \rho_{0} & = & |0\rangle \langle 0|,\;\;\;\rho_{1}=\left|J,0\rangle \langle J,0\right| \end{array}$$

as a candidate for the alphabet in a binary communication and compare it with Alphabet (1) with real z∈R, cf. Reference [3].

For a binary communication utilizing two states in either Equation (1) or Equation (10) as codewords, a receiver is faced with a decision of distinguishing which among two states has already been transmitted. The most natural quantifier of the distinguishability between two various states ρ and σ is the trace distance [19]:

(11) $$\begin{array}{ccc} D & = & \frac{1}{2}\mathrm{Tr}\left[\sqrt{{\left(\rho -\sigma \right)}^{2}}\right]. \end{array}$$

**Figure 1:**
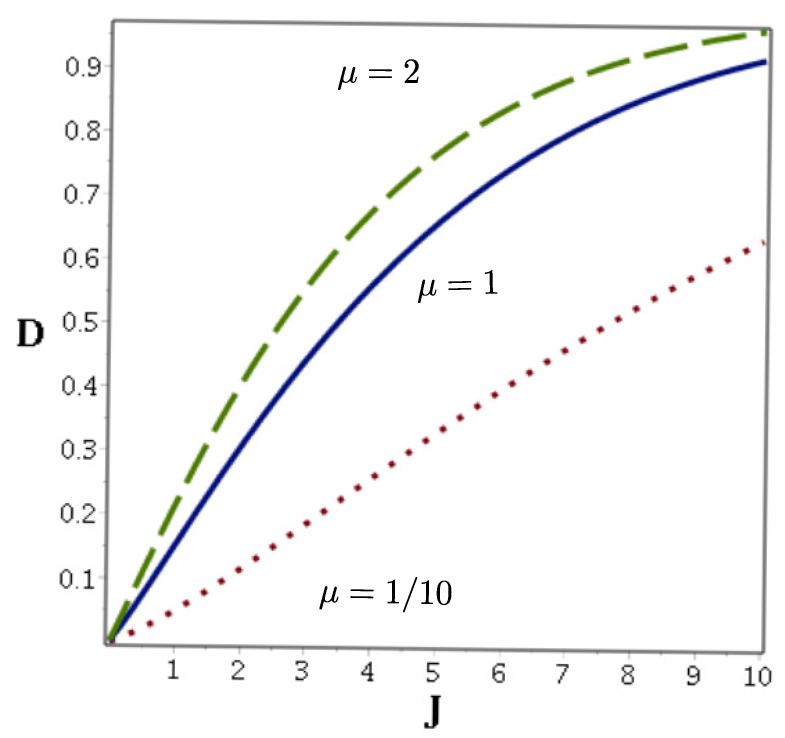
Trace distance between the Gazeau–Klauder coherent states |J,0〉 given by Equation (3) and the Perelomov coherent states $|z=\sqrt{J}\rangle$ depicted for selected values of the rescaled susceptibility μ.

The trace distance between the Gazeau–Klauder coherent state and the corresponding standard coherent state satisfying $z=\sqrt{J}$ can be calculated and the result is

(12) $$\begin{array}{c} D=\sqrt{1-{\left|F\right|}^{2}}, \end{array}$$

where

(13) $$\begin{array}{c} F=\frac{e^{-J/2}}{C(J)}\sum_{n=0}^{\infty }J^{n}\;\sqrt{\frac{\Gamma \left(\mu^{-1}\right)}{\mu^{n}\Gamma^{2}\left(n+1\right)\Gamma \left(\frac{\mu \;n+1}{\mu }\right)}}\; \end{array}$$

stands for fidelity [19]. Clearly, with increasing μ which quantifies the role played by polynomial nonlinearity of the Kerr medium in Equation (5) the trace distance becomes larger as presented in Figure 1. Nevertheless, for small values of *J*, the Gazeau–Kluder and Perelomov coherent states are hardly distinguishable.

Non-orthogonality of states $\rho_{0}$ and $\rho_{1}$ utilized as codewords in (binary) quantum communication becomes a natural source of error due to limited distinguishability of codewords. If one applies (resolving unity) POVM (positive-operator-valued measures) [19]

(14) $$\begin{array}{ccc} Id & = & \Pi_{0}+\Pi_{1} \end{array}$$

for a measurement of non-orthogonal states one arrives to two hypotheses $H_{0}$ and $H_{1}$ which need to be tested. According to $H_{0}$, the transmitted state is $\rho_{0}$ and according to $H_{1}$, the transmitted state is $\rho_{1}$. There is also the natural and unavoidable possibility of erroneous detection and choosing $H_{0}$ ($H_{1}$) if $\rho_{1}$ (respectively, $\rho_{0}$) arrives at a receiver. Such an opportunity can be formalized by quantities

(15) $$\begin{array}{ccc} p(H_{0}|\rho_{1}) & = & \mathrm{tr}\left[\Pi_{0}\rho_{1}\right],\;\;\;p\left(H_{1}|\rho_{0}\right)=\mathrm{tr}\left[\Pi_{1}\rho_{0}\right]. \end{array}$$

The receiver error probability becomes then

(16) $$\begin{array}{ccc} p[\Pi_{0},\Pi_{1}] & = & p_{0}\left(\rho_{0}\right)p\left(H_{1}|\rho_{0}\right)+p_{0}\left(\rho_{1}\right)p\left(H_{0}|\rho_{1}\right) \end{array}$$

with $p_{0}\left(\cdot \right)$ denoting the actual probability of transmission of a given state (1) and $p_{0}\left(\rho_{0}\right)+p_{0}\left(\rho_{1}\right)=1$. In quantum communication [20], there is a bound minimizing the receiver error (its lower bound)

(17) $$\begin{array}{ccc} P_{H} & = & \min_{\left\{\Pi_{0},\Pi_{1}\right\}}\;p\left[\Pi_{0},\Pi_{1}\right] \end{array}$$

known as the Helstrom bound [20] which, for a binary communication using the alphabet in Equation (10) reads [20,21]

(18) $$\begin{array}{ccc} P_{H} & = & \frac{1}{2}\left(1-\sqrt{1-4p_{0}\left(\rho_{0}\right)p_{0}\left(\rho_{1}\right){\left|\langle 0|J,0\rangle \right|}^{2}}\right). \end{array}$$

For the particular class of the Gazeau–Klauder coherent states studied in this paper, the Helstrom bound can be calculated explicitly:

(19) $$\begin{array}{ccc} P_{H} & = & \frac{1}{2}-\frac{1}{2}\;\sqrt{1-{\left(\left|I_{\frac{1-\mu }{\mu }}\left(2\;\sqrt{\frac{J}{\mu }}\right)\Gamma \left(\mu^{-1}\right){\left(\frac{J}{\mu }\right)}^{1/2\;\frac{-1+\mu }{\mu }}\right|\right)}^{-1}}. \end{array}$$

**Figure 2:**
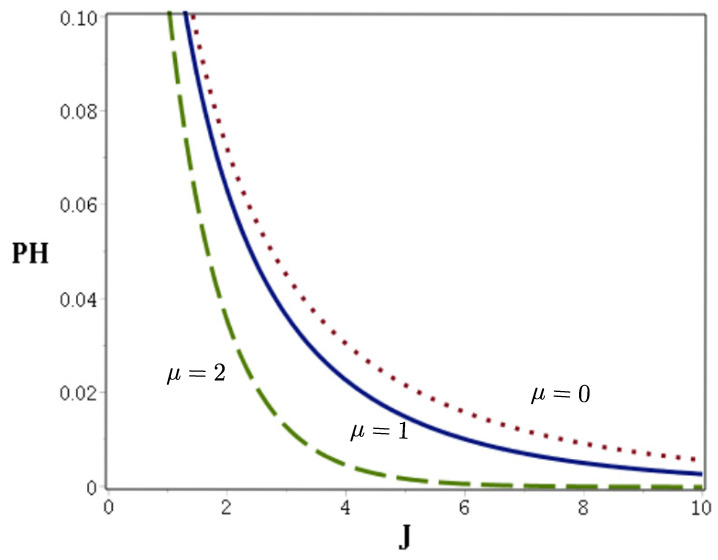
Helstrom bound $P_{H}$ given by Equation (19) depicted for selected values of μ. For the sake of clarity, the range of $P_{H}$ in the figure is limited to $P_{H}\leq 0.1$.

The Helstrom bound (19) for a binary communication with Gazeau–Klauder coherent states as letters of an alphabet for different values of μ in Equation (5) is presented in Figure 2.

Let us notice that for small values of J≤3 the Helstrom bound $P_{H}$ remains almost unaffected by nonlinearity even with relatively large amplitude μ≈2. With increasing *J* the effect of nonlinearity becomes more apparent resulting in lowering the value of the Helstrom bound, i.e., resulting in an advantageous smaller minimal probability of receiver error.

## 3. Discussion

Quantum communication implemented with quantum optical states and devices seems to be one of the most promising for future developments. Non-randomized coherent states are natural candidates for letters of an alphabet used in communication [2,3,4]. Creation and manipulation of such states do not require devices with a ‘hard’ nonlinearity. In this work we studied a binary alphabet consisting of two Gazeau–Klauder coherent states [8] as an alternative for a well studied choice of standard Perelomov coherent states. We utilize Gazeau–Klauder states calculated for a simplest nonlinear Kerr-type medium Equation (5) and we provide explicit analytic formulas for trace distance between Gazeau–Klauder and Perelomov states serving as codewords of the two alternative binary alphabets.

Despite that Gazeau–Klauder generalized coherent states [8] (used instead of the standard Perelomov) are harder in production [22], they can be, as we showed in this work, advantageous. At the cost of coping with a relatively well known Kerr-type nonlinear bosonic oscillator, present also beyond typical optical context [23], one gets a communication scheme with a smaller value of the Helstrom bound.

As coherent states with a non-randomized phase have recently attracted new attention [5] we believe that our analysis, despite its simplicity, can serve as a modest theoretical contribution for further practical developments utilizing Gazeau–Klauder coherent states in quantum communication and information processing and in a context of hybrid protocols [24].

## 4. Materials and Methods

Coherent states technique, quantum detection theory, quantum information with quantum optical implementations of quantum communication [4,6,8].
